# Real-world sex differences in treatment persistence and reasons for discontinuation in psoriatic arthritis patients: results from the German RABBIT-SpA register

**DOI:** 10.1186/s13075-025-03650-4

**Published:** 2025-10-02

**Authors:** Lisa Lindner, Anja Weiß, Andreas Reich, Christine Baumann, Frank Behrens, Xenofon Baraliakos, Anne C. Regierer

**Affiliations:** 1 German Rheumatology Research Center (DRFZ Berlin), Epidemiology and Health Services Research, Research Charitéplatz 1, 10117 Berlin, Germany; 2 Private Practice for Rheumatology, Plauen, Germany; 3 Fraunhofer Institute for Translational Medicine and Pharmacology ITMP, Frankfurt am Main, Germany; 4 Rheumatology Center Ruhrgebiet, Ruhr University Bochum, Herne, Germany

**Keywords:** Observational study, Psoriatic arthritis, Gender, Sex, Women, Men, First-line therapy, bDMARD, tsDMARD, Biologics, Therapy outcome, Patient-reported outcome

## Abstract

**Background:**

In psoriatic arthritis (PsA), growing evidence indicates sex-specific differences regarding clinical manifestation and treatment outcomes. Research has highlighted that females may be less likely to achieve treatment targets and are more prone to discontinuing therapy, though data on sex-specific adverse events is limited. This analysis investigates sex differences in treatment outcomes, persistence, discontinuation reasons, and adverse events during first-line b/tsDMARD therapy.

**Methods:**

In this analysis bionaïve patients with PsA from the RABBIT-SpA register were included at the start of their first b/tsDMARD. Therapy persistence was estimated using the Cox-regression adjusted for age. Descriptive analyses were used to examine and compare sex–specific differences on reasons for therapy discontinuation.

**Results:**

A total of 457 female patients and 343 male patients were included. Females exhibited more severe joint involvement and poorer patient-reported parameters, such as higher disease activity, more pain, and greater functional limitations. In contrast, males showed more pronounced skin involvement and a higher prevalence of nail psoriasis. Females had lower treatment persistence rates, both in the overall analysis of all first-line b/tsDMARDs and in subgroup analyses restricted to TNFi and IL17i therapies. At 12 months, 52% of females and 68% of males remained on their initial b/tsDMARD therapy. Notable sex differences were also observed in the reasons for therapy discontinuation: males more frequently discontinued due to lack of efficacy or remission, while females more often stopped treatment due to adverse events. Our safety analysis indicated that although female patients experienced a greater number of overall adverse events, males reported serious adverse events at twice the rate.

**Conclusions:**

Our findings underscore the need for sex-specific treatment strategies and more comprehensive research into biological and sociocultural factors influencing therapy persistence and reasons for discontinuation in real-world settings. Tailored treatment strategies are needed with regard to biologic therapy to overcome worse therapeutic outcomes in female patients with PsA.

**Clinical trial number:**

Not applicable.

**Supplementary Information:**

The online version contains supplementary material available at 10.1186/s13075-025-03650-4.

## Background

Psoriatic-Arthritis (PsA) is a chronic inflammatory disease that not only affects the joints but can also affect the skin, and entheses, with overall prevalence considered relatively equal between females and males [1]. However, several factors including hormonal influences, environmental and sociocultural factors, and genetic predispositions contribute to differences in disease expression and progression between the sexes [2].

Studies on PsA patients have shown sex-specific differences regarding clinical manifestation, disease impact and therapy response [3, 4]. Males typically have more axial and oligoarticular involvement, along with more severe skin manifestations and rapid radiographic damage. Females, on the other hand, are more likely to have polyarticular involvement, enthesitis and a higher disease burden including more functional limitations [5, 6].

Initial treatment of PsA includes conventional synthetic disease-modifying anti-rheumatic drugs (csDMARDs), while biologic and targeted synthetic DMARDs (b/tsDMARDs) such as tumor necrosis factor inhibitors (TNFi) and Interleukin-17 inhibitors (IL17i) are considered for patients who do not respond adequately to first-line options. The primary treatment objective is to achieve remission or at least minimal disease activity (MDA), aiming to prevent further joint damage and enhance patients’ overall well-being. The current EULAR (European Alliance of Associations for Rheumatology) recommendations stress the importance of a tailored treatment strategy, taking into account individual patients’ needs, comorbidities and preferences. To optimize individualized therapy, it is essential to recognize the influence of sex on these factors. Accordingly, research on the effect of sex on treatment choices, treatment efficacy, and treatment maintenance was prioritized in the recommendations for future research in PsA [7].

Current studies suggest that male sex may have a favorable impact on therapy persistence and outcome, with females less likely to achieve MDA [7–9]. Reasons for this disparity are multifaceted and may involve a combination of hormonal, biological, disease presentation and treatment-related factors. The specific mechanisms and reasons remain unclear.

The most common reasons for early therapy discontinuation in longitudinal observational studies include remission, lack of efficacy, and serious and non-serious adverse events (SAEs and AE) [10, 11]. However, a notable gap exists in sex-stratified data, particularly concerning adverse events during first-line b/tsDMARD therapy. Although some randomized trials have provided sex-stratified data on AEs [12], comprehensive observational studies with sex stratification remain limited.

The objectives of our analyses were to examine sex-specific differences in clinical parameters and first-line therapy persistence, as well as reasons for discontinuation. Additionally, we provide sex-stratified data on frequencies of SAEs and AEs occurring during the first-line b/tsDMARD therapy and leading to discontinuation.

## Patients and methods

### Data source

The RABBIT-SpA register is a German longitudinal observational cohort study focused on monitoring and evaluating the safety and effectiveness of b/tsDMARDs in patients with PsA in a real-world setting. Patients can be included by a rheumatologist with the start of a new b/tsDMARD or with a conventional systemic treatment e.g. csDMARD or NSAID (nonsteroidal anti-inflammatory drugs) [13]. Physicians and patients enter data using a web-based documentation system. Clinical and patient-reported data is collected through an electronic case report form (eCRF) at baseline, at three and six months post-enrolment, and subsequently at six-month intervals for a follow-up period of up to ten years [14].

### Patients

All PsA patients enrolled between October 2017 and database closure in March 2024 were included. For these analyses, only b/tsDMARD-naïve patients with PsA who initiated their first-line b/tsDMARD and who have a follow-up time of at least one year were selected (Supplementary Figure S1).

In our register, sex was reported as “female”, “male”, or “other”. Since no patient’s sex was reported as “other”, this study only refers to female and male patients.

While the distinction between sex and gender is well established in the social sciences, it remains a complex and evolving issue in medical research [15]. In this study, we use the term sex acknowledging that both biological and sociocultural factors may influence treatment outcomes, although the documentation of these variables may vary across study sites and may not consistently reflect this distinction.

### Statistical analyses and variables

#### Definition of variables

Characteristics at the beginning of first-line therapy of female and male participants were compared using appropriate descriptive statistics, including means, standard deviations, and percentages.

The physician eCRF contains data on treatment history and the current disease activity status and treatment regimen. Physicians record the onset of symptoms and the time of diagnosis. Disease duration is calculated in years since diagnosis, while diagnostic delay represents the time in years between onset of symptoms and diagnosis. Additional clinical parameters reported by the physician include dactylitis, axial involvement, and nail psoriasis, which are assessed using a binary yes or no response, enthesitis (SPARCC score including 16 entheses), swollen joint counts (66 joints, SJC-66), and tender joint counts (68 joints, TJC-68). The affected body surface area (BSA) is noted as a percentage and the C-Reactive Protein (CRP) value is provided as continuous value in mg/l. The DAS-28-CRP is calculated using 28 swollen and tender joint counts, CRP (mg/l), and patient global assessment. Physician global assessment is rated on a numeric rating scale (NRS 0–10). The DAPSA is a continuous measure focusing on joint involvement and providing granular information about disease activity [16]. Comorbidity data were obtained from the physician’s CRF, where comorbidities are documented using a checklist of predefined conditions (e.g., cardiovascular disease, renal disease, malignancies, depression) with the option to add further conditions in free-text fields. The Rheumatic Disease Comorbidity Index (RDCI) is used to quantify the burden of comorbid conditions in patients with rheumatic diseases [17] including PsA [18]. The index incorporates ten comorbidities (lung disease, myocardial infarction, stroke or other cardiovascular diseases, hypertension, fracture, depression, diabetes, cancer, and ulcers or stomach symptoms). These comorbidities, as documented by the physician, are assigned specific weights to generate a composite score ranging from 0 to 9. A higher value reflects a higher burden of comorbid conditions.

Patients provide data on sociodemographic characteristics and a variety of patient-reported outcome measures. The patient global assessment, pain, and sleep disturbance are rated on NRS 0–10. The WHO-5 Well-Being Index (WHO-5) is a widely used five-item instrument for screening depressive symptoms, yielding a total score from 0 to 100, with scores below 29 indicating moderate to severe depressive symptoms. Higher scores reflect greater well-being [19, 20]. The Dermatology Life Quality Index (DLQI) is reported annually by the patients to assess the impact of dermatological conditions on their quality of life. The Health Assessment Questionnaire (HAQ) is administered at each time point to evaluate the patients’ functional ability and the extent of disability related to their condition.

#### Therapy persistence rate

The Kaplan-Meier method was utilized to estimate therapy persistence to visualize the time-to-treatment discontinuation of the first-line b/tsDMARD therapy for the two groups of interest. One therapy episode was defined as an episode of the same active substance that has not been paused for more than 90 days. Patients who continued therapy after database close or were lost-to-follow up were censored.

To analyze the direct effect of sex on therapy persistence, we used a minimally adjusted model. Age was included as covariate, as it may influence therapy persistence independently, through factors such as comorbidities, treatment tolerability, or healthcare-seeking behavior, but is not considered a mediator in the relationship between sex and therapy persistence. Additional covariates that could potentially act as mediators, such as disease severity or other sociodemographic factors, were deliberately excluded to avoid attenuating or obscuring the direct effect of sex [21, 22]. By adjusting for age only, we aimed to isolate the specific contribution of sex to therapy persistence, without introducing other variables that might blur this relationship. The age-adjusted analysis was performed using a Cox Regression model.

#### Reasons for discontinuation

Physician-reported reasons for discontinuation were categorized into adverse events, lack of efficacy, remission and other reasons. For all patients who stopped their first-line therapy during observation proportion of reasons for discontinuation were analyzed.

#### Adverse events analysis

To enhance clarity, SAEs and AEs are collectively referred to as events unless otherwise specified and needed.

In this event analysis we only included patients for whom the reason to discontinue was reported as “(serious) adverse event”. The specific event(s) that led to therapy discontinuation cannot definitively be identified, due to the way events are reported in the register, because physicians are not explicitly asked to specify which specific event or a combination of events was the reason for discontinuation.

Therefore, to identify an event as a treatment-related reason for discontinuation, we used the following assumptions: The primary criterion to classify an event, as either treatment-related or non-treatment-related, was the causal relationship to the therapy indicated by the physician as ‘definite,’ ‘probable,’ or ‘possible’. Secondly, if no information on the causal relationship was provided or it was indicating an ‘unlikely’, ‘no causal’ or ‘unknown’ relationship, the event was selected time-based. This means if the event occurred no more than 90 days before and 14 days after the date of therapy discontinuation, it was eligible for selection and therefore matching to the therapy episode. The risk windows for neoplasms and malignancies were extended to the end of observation time. If an event could not be classified as either having a causal or time-based relationship to the first-line therapy, it was considered out of range and excluded from further event analysis.

## Results

### Patients characteristics: demographics, disease variables and patient-reported outcomes

Table 1 presents the characteristics at start of first-line therapy of 800 patients with PsA, stratified by female sex (*n* = 457) and male sex (*n* = 343). The mean age was 52.3 years in females, while males were slightly younger. A higher proportion of females reported being current smokers and they were more frequently obese. Disease duration for joints and skin involvement was similar for both sexes. Females exhibited a longer diagnostic delay for joint and skin involvement compared to males. And they had less BSA affected. Nail psoriasis and dactylitis were more common among males. The proportion of patients with ≥ 5 mg/l CRP was similar between the sexes.

**Table 1:** Sex-Stratified Patient Characteristics in RABBIT-SpA

Patient Characteristics	Females (*n* = 457)	Males (*n* = 343)	Total (*n* = 800)	Missings In %
**Sociodemographic Factors**				
Age, years, Mean (SD)	52.3 (12.7)	49.9 (13)	51.3 (12.9)	0
BMI, kg/m^2^, Mean (SD)	28.8 (6.5)	28.4 (4.7)	28.7 (5.8)	1
BMI > 30, kg/m^2^, Yes, n (%)	166 (37)	109 (32)	275 (35)	
Current Smoker, Yes, n (%)	144 (36)	71 (24)	215 (31)	14
**Physician-Reported Measure**				
Disease Duration, years				
Joints, Mean (SD)	4.9 (7.0)	4.5 (6.3)	4.7 (6.7)	10
Skin, Mean (SD)	12.9 (14.5)	12.8 (13.8)	12.9 (14.2)	33
Diagnostic Delay, years				
Joints, Mean (SD)	3.1 (6.0)	2.1 (4.4)	2.6 (5.4)	10
Skin, Mean (SD)	3.5 (8.4)	2.2 (6.3)	2.9 (7.6)	36
Dactylitis, Yes, n (%)	76 (17)	70 (21)	146 (18)	1
Axial Involvement, Yes, n (%)	89 (20)	63 (19)	152 (19)	1
Nail Psoriasis, Yes, n (%)	152 (34)	160 (47)	312 (39)	1
BSA, Mean (SD)	6.1 (11.6)	10.7 (16)	8 (13.8)	3
CRP, mg/L				6
Mean (SD)	6.3 (10.2)	7 (12.5)	6.6 (11.2)	
≥ 5 mg/l, n (%)	162 (38)	128 (39)	290 (39)	
HLA-B27, Positive, n (%)	45 (16)	46 (19)	91 (17)	35
Enthesitis, Yes, n (%)	122 (27)	69 (20)	191 (24)	1
Mean (SD)	0.8 (2.0)	0.5 (1.3)	0.7 (1.7)	
Tender Joints (TJC-68), Yes, n (%)	387 (85)	270 (79)	657 (82)	0
Mean (SD)	6.8 (7.2)	5.3 (7.0)	6.1 (7.1)	
No Affected Joints, n (%)	69 (15)	72 (21)	141 (18)	
≥ 5 affected joints, n (%)	226 (50)	122 (36)	348 (44)	
Swollen Joints (SJC-66), Yes, n (%)	315 (69)	216 (63)	531 (67)	0
Mean (SD)	3.3 (4.3)	2.8 (3.9)	3.1 (4.2)	
No affected joints, n (%)	141 (31)	126 (37)	267 (33)	
≥ 5 affected joints, n (%)	107 (23)	67 (20)	174 (22)	
Physician Global Disease Activity*, Mean (SD)	5.1 (1.9)	4.7 (2)	4.9 (2)	2
DAPSA, Mean (SD)	22.9 (12.7)	19 (12.3)	21.2 (12.6)	16
REM (0–4), n (%)	11 (3)	18 (6)	29 (4)	
LDA (5–14), n (%)	82 (21)	93 (32)	175 (26)	
MoDA (15–28), n (%)	182 (48)	135 (46)	317 (47)	
HDA (> 28), n (%)	108 (28)	46 (16)	154 (23)	
DAS-28-CRP, Mean (SD)	3.6 (1.1)	3.2 (1.1)	3.4 (1.1)	16
No. of comorbidities (0–47), Mean (SD)	1.9 (2.0)	1.7 (2.2)	1.8 (2.1)	
≥ 3 comorbidities, n (%)	138 (30)	78 (23)	216 (27)	
RDCI (0–9), Mean (SD)	0.9 (1.1)	0.7 (1.0)	0.8 (1.1)	0
Depression as comorbidity, n (%)	61 (13)	19 (6)	80 (10)	
Fibromyalgia as comorbidity, n (%)	23 (5)	2 (1)	25 (3)	
**Patient-Reported Outcomes**				
Patient Global*, Mean (SD)	5.8 (2.3)	5.0 (2.5)	5.5 (2.5)	10
Patient Pain*, Mean (SD)	5.8 (2.3)	4.7 (2.5)	5.3 (2.4)	10
Patient Sleep Disturbance*, Mean (SD)	5.5 (3)	3.9 (2.9)	4.8 (3)	12
WHO5 (0-100), Mean (SD)	42.0 (22.1)	52.1 (22.3)	46.3 (22.8)	13
Moderate/Severe (< 29), n (%)	133 (33)	56 (19)	189 (27)	
DLQI, Mean (SD)	5.2 (6.2)	5.1 (5.7)	5.1 (6)	13
HAQ, Mean (SD)	1.0 (0.6)	0.6 (0.6)	0.9 (0.6)	11

BSA: Body surface area; DAPSA: Disease Activity in Psoriatic Arthritis; REM: Remission; LDA: Low Disease Activity; MoDA: Moderate Disease Activity, HDA: High Disease Activity; RDCI: Rheumatic Disease Comorbidity Index; WHO-5: World Health Organization-Five Well-Being Index; DLQI: Dermatology Life Quality Index; HAQ: Health Assessment Questionnaire. ^*^NRS-Scale (0–10)

The average number of SJC-66 and TJC-68 was higher in females, as well as the proportion of patients with ≥ 5 affected joints. Females also had a higher proportion of enthesitis and more enthesitis sites. Females had a slightly higher mean physician global disease activity score, DAPSA and DAS-28-CRP compared to males.

Female patients reported higher values in most patient-related outcome measures including patient global, pain, sleep disturbance, functional status, and well-being. The mean DLQI score was comparable for both sexes.

### Treatments

Table 2 presents the proportion of first-line b/tsDMARDs, concomitant treatments, and therapy continuation after one year, stratified by sex. Among first-line b/tsDMARD therapies, TNFi was the most commonly used treatment in both females and males, prescribed to 54% of females and 57% of males. The second most frequently prescribed therapy was IL17i, administered to 26% of females and 31% of males.

NSAID use was reported for 43% of females compared to 35% of males. Glucocorticoids were prescribed in 32% of the overall population, with a higher use among females. Additionally, both non-opioid and opioid analgesics were more commonly used in females.

**Table 2:** First-Line Therapies and Concomitant Therapies at Treatment Initiation in Females and Males with PsA

	Females (*n* = 457)	Males (*n* = 343)	Total (*n* = 800)
**First-Line Therapy, n (%)**			
First-Line TNFi	247 (54)	197 (57)	444 (56)
First-Line IL17i	119 (26)	107 (31)	226 (28)
First-Line Other Modes of Action	91 (20)	39 (11)	130 (16)
IL23i	13 (14)	3 (8)	16 (12)
IL12i/IL23i	13 (14)	9 (23)	22 (17)
IL6i	1 (1)	0 (0)	1 (1)
JAKi	19 (21)	8 (21)	27 (21)
PDE4-Inhibitor	44 (48)	19 (49)	63 (48)
T-Cell Costimulation Inhibitors	1 (1)	0 (0)	1 (1)
**Concomitant Therapies, n (%)**			
Current Glucocorticoids, n (%)	156 (34)	99 (29)	255 (32)
Glucocorticoid Dose, mean (SD)	7 (4.8)	8.8 (7.2)	7.7 (5.9)
Current Non-Opioid Analgesics Therapy	87 (26)	47 (19)	134 (23)
Current Opioid Therapy	30 (11)	17 (8)	47 (9)
Current NSAID Therapy	194 (43)	119 (35)	313 (39)
Current csDMARD Therapy	197 (43)	154 (45)	351 (44)

### Persistence rates

**Fig. 1:**
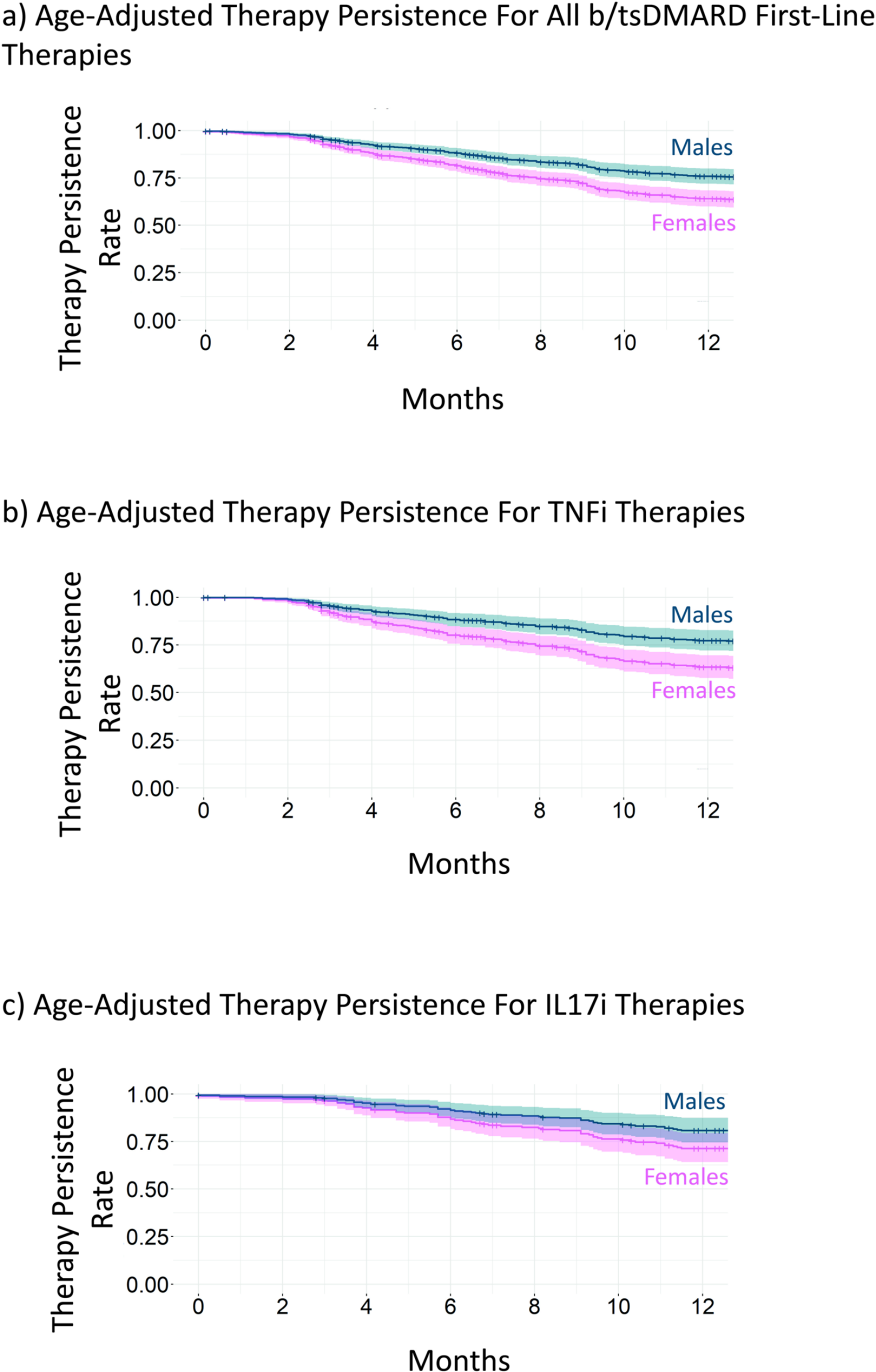
Therapy Persistence Rates per Group Across All Therapies, TNFi and IL17i

Figure 1 presents the age-adjusted therapy persistence rates across all therapies (1a) (including TNFi, IL17i and other modes of action) as well as specifically for TNFi (1b) and IL17i (1c). Overall, 59% of patients remained on their first-line b/tsDMARD therapy after one year of observation, with 52% of females and 68% of males continuing their initial treatment (HR: 0.62 [0.50–0.76], *p* = 0.000). Males demonstrated higher persistence rates across all therapies after adjustment, as well as for TNFi (HR: 0.56 [0.42–0.76], *p* = 0.000) and IL17i (HR: 0.63 [0.42–0.96], *p* = 0.030) in particular.

### Reason for therapy discontinuation

Of 800 patients, 328 patients discontinued their first-line treatment during follow-up within 12 months. The reason for discontinuation was reported in 213 patients (65%) (Table 3). The most common reasons were lack of efficacy and AEs. A higher proportion of males discontinued therapy due to lack of efficacy. In contrast, more females discontinued due to AEs (22%) compared to males (13%). Remission was reported as a reason for discontinuation in 2% of patients. Both sexes discontinued therapy for other unspecified reasons at an equal rate, including for example non-compliance, patient decision, and other medical interventions unrelated to the rheumatic disease.

**Table 3:** Reasons for Therapy Discontinuation as Recorded by the Treating Physician

	Females (*n* = 218)	Males (*n* = 110)	Total (*n* = 328)
**Reason for Therapy Discontinuation**			
(Serious) Adverse Events, n (%)	49 (22)	14 (13)	63 (19)
Lack of Efficacy, n (%)	83 (38)	45 (41)	128 (39)
Remission, n (%)	1 (0)	4 (4)	5 (2)
Other Reason^a^, n (%)	12 (6)	5 (5)	17 (5)
Unknown (%)	73 (33)	42 (38)	115 (35)

^a^e.g. non-compliance, patient decision, other medical interventions

### Serious and non-serious adverse events

In total, 171 events occurred in 63 patients for whom the reason to discontinue was reported as “(serious) adverse event” (Table 4). The overall number of events was 127 in females and 44 in male patients. Out of 127 events, 10 were classified as SAE and 117 as AE in females. In male patients, 11 were SAEs and 33 were AEs. For females 91 and for males 30 events could either be related to a causal or time-based relationship. A total of fifty events could not be identified as causal or time-based relationship and were excluded from further analysis. Out of all treatment related events, more events were reported as serious in males (20%) than in females (8%).

**Table 4:** Identification of Events as Treatment-Related Reasons for Therapy Discontinuation

	Females	Males	Total
Patients Who Discontinued Due to an Event and Had (S)AE Reported	49	14	63
**Identification of Events as Treatment-Related Reasons for Discontinuation**			
Overall No. of Events Occurred, n	127	44	171
Causal relationship to therapy^a^	61 (48)	17 (39)	78 (45)
Time-based relationship^b^	30 (24)	13 (29)	43 (25)
Event out of range or missing^c^	36 (28)	14 (32)	50 (30)
No. of Treatment-Related Events, n	91	30	121
Thereof SAEs	7 (8)	6 (20)	13 (11)
Thereof AEs	84 (92)	24 (80)	108 (89)

All data presented as n (%)

^a^Indicated by physician as ‘definite,’ ‘probable,’ or ‘possible’

^b^Events occurring no more than 90 days before or 14 days after the date of therapy discontinuation where eligible for selection and therefore matching to the therapy episode

^c^If an event could not be classified as either having a causal or time-based relationship to the first-line therapy or was missing, it was considered out of range and excluded from further analysis

In Table 5, the treatment related events are further classified. The most common events were infections, neurologic events, and other events, which were not specifically categorized. A list of preferred terms (within the MEdDRA hierarchy) can be found in the supplement (Supplementary Table S2).

In fifteen females (14 had AEs and 1 had a SAE) twenty-two infections (21 AEs and 1 SAE) and in 3 males (2 had AEs and 1 had a SAE) four infections (3 AEs and 1 SAE) occurred. The most common infections were influenza, oral herpes, nasopharyngitis, and cystitis. One serious flare event occurred in one female. One serious cardiologic event was reported for one male patient, which was an intracardiac thrombus. Neurologic/psychiatric events were reported in nine females (12 AEs) and three males (3 AEs) but no serious adverse events occurred in this category. Neurologic/psychiatric events include, among others, depression, headache, dizziness, and mood swings. One female had a haematologic AE, which was leucopenia. A benign neoplasia event was documented in three females. These were pulmonary mass, papilloma and uterine leiomyoma.

The most frequently documented events were labeled as “other events”, and included for example allergic conditions, diarrhea, skin disorders, alopecia, other musculoskeletal disorders, and pruritus.

**Table 5:** Treatment-Related Serious (SAE) and Non-Serious Adverse Events (AE)

	Females (*n* = 49)		Males (*n* = 14)	
n event / n patient*	*Non-serious adverse events (AE)*	*Serious adverse events* *(SAE)*	*Non-serious adverse events (AE)*	*Serious adverse events* *(SAE)*
Infection	21/14	1/1	3/2	1/1
Flare	0/0	1/1	0/0	0/0
Cardiologic Events	0/0	0/0	0/0	1/1
Neurologic/Psychiatric Events	12/9	0/0	3/3	0/0
Hematologic Events	1/1	0/0	0/0	0/0
Neoplasia	3/3	0/0	0/0	0/0
Other Events	47/29	5/3	18/11	4/2
Total	84/56	7/5	24/16	6/4

*n event / n patient: represents the ratio of the number of events that occurred and the number of patients who experienced an event. Patients may be represented in multiple categories

## Discussion

This study investigated the differences between female and male patients with PsA, regarding variations in disease characteristics, therapy persistence, and reasons for discontinuation. Furthermore, we placed a special focus on sex differences in treatment-related adverse events in a real-world setting.

We analyzed 800 patients starting their first b/tsDMARD therapy. We had a higher number of female patients in the cohort, and they were, on average, two years older than males. Females had a one year longer diagnostic delay, exhibited worse health-related outcomes, and showed higher musculoskeletal disease activity at the start of treatment while males presented more severe skin involvement. These findings align with previously presented data from systematic reviews on observational studies and randomized clinical trials [3, 23]. While males had a higher mean BSA affected by psoriasis, the disease burden, as measured by the DLQI, was similar for both sexes, which is consistent with the findings of a scoping review by dermatologists [24].

TNFi was the most frequently prescribed b/tsDMARD among both sexes, followed by IL17i, which were more commonly prescribed to males, while females more often received IL23i. As IL17i are particularly effective in treating skin manifestations [25], the more frequent prescription of IL17i in males may be due to the greater skin involvement at start of the first line therapy in our cohort. Other modes of action were comparable in frequency. The frequent use of glucocorticoids, even more common in females than in males, does not correspond to current treatment recommendations [26].

Regarding treatment, females had lower persistence rates for both TNFi and IL17i in our study. This corresponds to results from the Danish DANBIO register, where males also remained on TNFi first-line therapy for a longer duration [27]. Another recent study analyzing pooled data from 13 European registries within the EuroSpA collaboration found that females were more likely to discontinue TNFi therapy within a two-year period with a higher likelihood of discontinuation due to insufficient efficacy or side effects [10]. Further supporting this, an Italian cohort study observed that females were more likely to discontinue anti-TNFi treatments earlier than males, although smaller sex differences were noted for IL17i and IL12/23i therapies. However, the smaller sample sizes in these groups limit the generalizability of these findings [28]. Similarly, a multinational prospective cohort study by Van Kuijl et al. demonstrated that females who started ustekinumab, regardless of the therapy line, showed lower persistence [29].

A trend observed across multiple studies suggests that females experience higher rates of discontinuation due to side effects, particularly with TNFi and IL17i therapies. This observation is further supported by a meta-analysis of randomized controlled trials (RCTs), which found lower response rates and higher discontinuation rates when treated with TNFi and IL17i in females. On the other hand, for Janus kinase inhibitors (JAKi), the efficacy between the sexes was similar, but females experienced a higher incidence of adverse events, which could contribute to their lower therapy persistence [23].

These sex-based differences in therapy persistence may be further elucidated by examining the reason for therapy discontinuation, where we found notable differences.

While the most common reason for discontinuation among both sexes was lack of efficacy, interestingly, males were three times more likely to discontinue therapy due to remission, further supporting the findings related to treatment response and effectiveness. Females, on the other hand, were more likely to discontinue treatment because of safety events. Our safety data showed that, during the initial b/tsDMARD intervention, females had more safety events overall, but SAEs were reported twice as often in males. This finding aligns with a worldwide study on differences in reporting drug events in which male reports were more frequently classified as SAE [30]. The reasons are mainly unclear and could be related to actual physiological differences in therapy response or gendered reporting practices and healthcare-seeking behaviors. For example, women utilize healthcare program offerings more regularly [31]. They also tend to report more events than men, which can possibly lead to an earlier detection and intervention on these potential serious impairments.

As expected, the most common treatment-related events overall were infections, neurologic/psychiatric and other events, with more non-serious events reported in females. Most of the infectious events were attributable to flu-like infections. In a study on patients with psoriasis receiving bDMARDs, females had significantly more fungal and herpes simplex infections compared to males [32]. In this study, the authors report that females experienced more side effects and had a lower overall satisfaction rate, which they suggest may explain the lower therapy persistence rates observed in females. A post-hoc analysis of a phase 3 trial with tofacitinib showed similar proportions among both sexes who experienced AEs. However, females treated for up to 12 months had a higher incidence of SAEs [23]. Gosselt et al. also found that compared to men, women report more and a wider variety of adverse drug reactions (ADR) in patients receiving adalimumab and etanercept [33]. They also examined the burden of ADR and found that there is no difference in reported burden between women and men. This could indicate, that although women are more likely to report events, they do not necessarily experience them in a more severe or burdensome way than men, which might simply reflect more frequent reporting rather than actual differences in severity or overall experience of ADR.

Our findings underscore significant sex-specific differences among patients with PsA in terms of baseline characteristics, treatment outcomes, and persistence with therapy. Moreover, we provide novel insights into the underlying reasons for treatment discontinuation, revealing distinct patterns between female and male patients. Additionally, our analysis highlights sex-based disparities in the nature and incidence of adverse events.

### Strengths and limitations

A strength of our study is the large prospective, observational, real-world data source providing a close monitoring of efficacy and safety under all approved b/tsDMARD strategies within predefined follow up intervals. The sample size enabled sex-stratified analyses on reasons for therapy discontinuation, along with detailed information on adverse events.

Limitations: Due to the observational nature of the registry, causal inferences cannot be drawn from our findings. As we only capture sex as a simple variable, we cannot separate sex and gender but recognize that both biological processes and gender roles likely influence outcomes.

Due to the design of the questionnaires, we could not determine the exact event that led to therapy discontinuation, but had to make assumptions and assigned the events primarily via temporal context. In 35% of therapy discontinuations, the reason for discontinuation was not documented. This proportion of missing information may have influenced the observed distribution of discontinuation causes between sexes and should be considered when interpreting these results. While we adjusted only for age to retain the total observed effect of sex, other potential confounders (e.g., comorbidities, smoking, concomitant drugs) were not included in the models. Thus, residual confounding may contribute to the observed sex differences and our findings should be interpreted accordingly. Differences in reporting behavior between sexes may have influenced our findings. In particular, females may be more likely to report less severe AE, potentially inflating AE-related discontinuation rates compared to males. This possible reporting bias could partly explain the observed sex differences and should be considered when interpreting the results.

### Conclusion

This study observed sex differences in PsA, with women showing higher baseline disease activity and musculoskeletal burden, but lower therapy persistence, particularly with TNFi and IL17i therapies. Male patients were more likely to discontinue treatment due to remission, while females discontinued more often due to side effects, highlighting a key difference in treatment-related adverse events.

These findings emphasize the need for sex-specific strategies in managing PsA to improve therapeutic outcomes and patient satisfaction.

More nuanced research into both biological and sociocultural factors influencing therapy outcomes in PsA is needed, especially in real-world settings. Observational studies provide valuable insights but the lack of standardized reporting of sex data limits the ability to draw definitive conclusions. Capturing sex-related variables across all types of studies is an important future direction to broaden our knowledge and deepen our understanding of the underlying factors contributing to observed disparities.

## Supplementary Information

Below is the link to the electronic supplementary material.

Supplementary Material 1

## Data Availability

The data that support the findings of this study are available from German Rheumatology Research Center but restrictions apply to the availability of these data, which were used under license for the current study, and so are not publicly available. Data are however available from the authors upon reasonable request and with permission of the German Rheumatology Research Center.
