# Supplementary material for: Real-world sex differences in treatment persistence and reasons for discontinuation in psoriatic arthritis patients: results from the German RABBIT-SpA register
Source: Arthritis Res Ther. 2025 Oct 2;27:188. doi: 10.1186/s13075-025-03650-4 (PMC12490096; doi:10.1186/s13075-025-03650-4)
Supplement: Supplementary file 1 — Supplementary Material 1 [file 13075_2025_3650_MOESM1_ESM.docx]

**Supplementary Table and Figure**
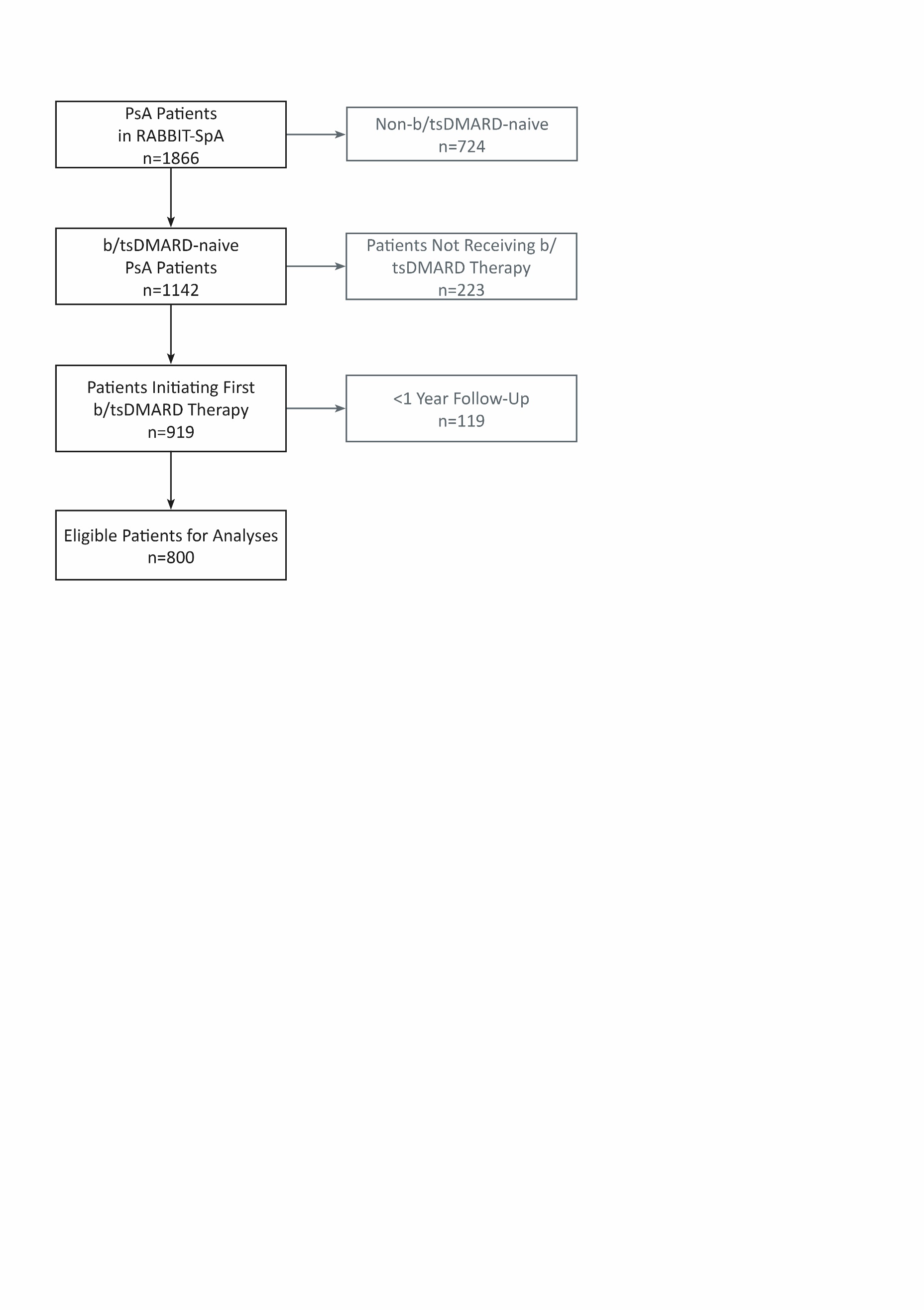


Supplementary Figure S1: Patients Eligibility Flowchart.

*Supplementary Table S2: Preferred MedDRA Terms Representing Serious (SAE) and Non-Serious Adverse Events (AE) Per Sex.*

|  | **Females** | | **Males** | | **Total** |
| --- | --- | --- | --- | --- | --- |
| **MedDRA Preferred Term** | **AE** | **SAE** | **AE** | **SAE** |  |
| Abdominal abscess |  |  | 1 |  | 1 |
| Abdominal discomfort | 2 |  |  |  | 2 |
| Abdominal pain | 1 |  |  |  | 1 |
| Abscess |  |  | 1 |  | 1 |
| Alopecia | 1 |  |  |  | 1 |
| Alopecia areata | 2 |  |  |  | 2 |
| Amaurosis fugax |  |  | 1 |  | 1 |
| Aphthous ulcer | 1 |  |  |  | 1 |
| Arthroscopic surgery |  |  |  | 1 | 1 |
| Asthma | 1 |  |  |  | 1 |
| Back pain |  |  | 1 |  | 1 |
| Bronchitis | 1 |  |  |  | 1 |
| Collagen disorder |  | 1 |  |  | 1 |
| Complex regional pain syndrome | 1 |  |  |  | 1 |
| Cystitis | 2 |  |  |  | 2 |
| Depression | 3 |  |  |  | 3 |
| Dermatitis allergic | 1 |  |  |  | 1 |
| Diarrhoea | 6 |  | 3 |  | 9 |
| Dizziness | 1 |  | 1 |  | 2 |
| Drug eruption | 1 |  |  |  | 1 |
| Drug intolerance |  |  | 1 |  | 1 |
| Dry mouth | 1 |  |  |  | 1 |
| Epistaxis | 1 |  |  |  | 1 |
| Eye pruritus | 1 |  |  |  | 1 |
| Fall |  |  | 1 |  | 1 |
| Fatigue | 1 |  |  |  | 1 |
| Feeling hot | 1 |  |  |  | 1 |
| Fungal infection | 1 |  |  |  | 1 |
| Gastrointestinal infection | 1 |  |  |  | 1 |
| Gestational diabetes |  | 1 |  |  | 1 |
| Gingivitis | 1 |  |  |  | 1 |
| Headache | 2 |  | 1 |  | 3 |
| Hepatic enzyme increased | 1 |  | 1 |  | 2 |
| Hepatitis E | 1 |  |  |  | 1 |
| Herpes simplex | 2 |  |  |  | 2 |
| Hip arthroplasty |  |  |  | 1 | 1 |
| Hypertension |  |  | 1 |  | 1 |
| Impaired healing |  |  | 1 | 1 | 2 |
| Influenza |  | 1 |  |  | 1 |
| Influenza like illness | 2 |  | 1 |  | 3 |
| Injection site erythema | 1 |  |  |  | 1 |
| Injection site pain | 1 |  |  |  | 1 |
| Injection site pruritus | 1 |  |  |  | 1 |
| Injection site reaction |  |  | 1 |  | 1 |
| Interstitial lung disease | 1 |  |  |  | 1 |
| Intervertebral disc protrusion |  |  | 1 |  | 1 |
| Intracardiac thrombus |  |  |  | 1 | 1 |
| Leukopenia | 1 |  |  |  | 1 |
| Mood swings | 2 |  |  |  | 2 |
| Muscle spasms | 3 |  |  |  | 3 |
| Myalgia | 1 |  |  |  | 1 |
| Nasal herpes | 1 |  |  |  | 1 |
| Nasopharyngitis | 2 |  | 1 |  | 3 |
| Nausea | 2 |  |  |  | 2 |
| Oesophagitis | 1 |  |  |  | 1 |
| Oral candidiasis | 1 |  |  |  | 1 |
| Oral herpes | 3 |  |  |  | 3 |
| Osteoarthritis |  |  |  | 1 | 1 |
| Osteoporosis |  | 1 |  |  | 1 |
| Papilloma | 1 |  |  |  | 1 |
| Pharyngitis | 1 |  |  |  | 1 |
| Pollakiuria | 1 |  |  |  | 1 |
| Polyneuropathy | 1 |  |  |  | 1 |
| Pruritus | 3 |  |  |  | 3 |
| Psoriasis | 1 | 1 | 1 |  | 3 |
| Psoriatic arthropathy |  | 1 |  |  | 1 |
| Pulmonary fibrosis |  | 1 |  |  | 1 |
| Pulmonary mass | 1 |  |  |  | 1 |
| Rash | 1 |  | 2 |  | 3 |
| Respiratory tract infection | 2 |  |  |  | 2 |
| Sciatica | 1 |  |  |  | 1 |
| Sensory level abnormal | 1 |  |  |  | 1 |
| Skin discolouration |  |  | 1 |  | 1 |
| Stomatitis | 1 |  |  |  | 1 |
| Tendonitis | 1 |  |  |  | 1 |
| Transaminases increased |  |  | 1 |  | 1 |
| Tremor | 1 |  |  |  | 1 |
| Urinary tract infection | 1 |  |  | 1 | 2 |
| Uterine leiomyoma | 1 |  |  |  | 1 |
| Vomiting | 1 |  |  |  | 1 |
| Weight increased | 3 |  |  |  | 3 |
| Wrist fracture |  |  | 1 |  | 1 |
| **Total** | **84** | **7** | **24** | **6** | **121** |
